# Post-Pandemic Perspectives: Willingness, Risk Perception and Factors Influencing COVID-19 Booster Vaccine Uptake Among Thai Healthcare Workers and Vulnerable Populations

**DOI:** 10.3390/vaccines12121381

**Published:** 2024-12-07

**Authors:** Amornphat Kitro, Wachiranun Sirikul, Chanachai Polpitakchai, Jinjuta Panumasvivat, Ranchana Yamsiri, Pacharee Tasena, Chutima Punyaphab, Chaiy Rungsiyakull, Ratana Sapbamrer, Penprapa Siviroj, Kriengkrai Srithanaviboonchai

**Affiliations:** 1 Department of Community Medicine, Faculty of Medicine, Chiang Mai University, Chiang Mai 50200, Thailand; amornphat.kit@cmu.ac.th (A.K.);; 2 Environmental and Occupational Medicine Excellence Center, Faculty of Medicine, Chiang Mai University, Chiang Mai 50200, Thailand; 3 Center of Data Analytics and Knowledge Synthesis for Health Care, Chiang Mai University, Chiang Mai 50200, Thailand; 4 Health Promotion Unit, Maharaj Nakorn Chiang Mai, Faculty of Medicine, Chiang Mai University, Chiang Mai 50200, Thailand; 5 Department of Mechanical Engineering, Faculty of Engineering, Chiang Mai University, Chiang Mai 50200, Thailand; 6 Research Institute for Health Sciences, Chiang Mai University, Chiang Mai 50200, Thailand

**Keywords:** COVID-19 vaccine post-pandemic, risk perception, Thailand, healthcare workers, high-risk groups

## Abstract

Background: The emergence of new COVID-19 variants continues to affect healthcare workers (HCWs) and vulnerable populations in the post-pandemic era. This study aims to assess the willingness, perceptions, and factors associated with booster COVID-19 vaccine uptake in this context. Methods: A cross-sectional study was conducted between October 2023 and May 2024 among Thai adults (>20 years old) in Chiang Mai, Northern Thailand. Participants included HCWs and patients with chronic medical conditions. People who had received a monovalent XBB-derived booster vaccine were excluded. Results: Data related to a total of 811 participants were analyzed, with 328 from the vulnerable group and 483 HCWs. Willingness to receive the booster was similar in both groups (43.3% in HCWs, 45.0% in the vulnerable group). Low-risk perception (59.6%–83.5%), minimal impact on daily life (60.4%–62.9%), and doubts about booster efficacy (75.9%–81.4%) were prevalent negative thoughts toward the booster. Having received a flu vaccine (aOR 2.1), concerns about the impact on life of COVID-19 (aOR 1.8), and beliefs in booster safety (aOR 1.8) and vaccine effectiveness against severe disease (aOR 2.7) were associated with increased willingness. Conclusions: Only 44% of participants were willing to receive a COVID-19 booster. Policymakers can use these insights to develop strategies to increase vaccine uptake in the post-pandemic era.

## 1. Introduction

COVID-19 emerged as a global pandemic and a critical international public health emergency between 2020 and 2023, profoundly impacting individual health, communities, and the social and economic climate worldwide [1]. According to the latest World Health Organization (WHO) epidemiological update in October 2024, the cumulative global case count reached 776 million, with more than 7 million deaths [2]. However, this number may underestimate the true number due to reduced testing, sequencing, and reporting capacities. Surveillance of viral load in wastewater suggests the actual burden could be clinically underestimated by between two and nineteen times [3,4,5]. Until recently, there was still a seasonal surge causing morbidity and mortality from new variants, immune waning from vaccination or infection, and reduced adherence to personal protective measures [6,7,8]. Omicron variants are currently spreading with many mutations, including variants of interest (VOIs) BA.2.86 and JN.1 and variants under monitoring (VUMs)—JN.1.7, JN.1.18, KP.2, KP.3, KP.3.1.1, LB.1, and XEC. All variants spread easily [2].

COVID-19 vaccines currently available are typically around 60% effective in preventing the disease and can reduce hospitalizations, deaths, and instances of long COVID-19 by about 80% [9,10]. However, the effectiveness of vaccines tends to decrease over time, usually within 3–6 months after vaccination. Especially among older individuals with chronic illnesses, vaccine effectiveness drops from 87% one month after vaccination to 47% after five months [11,12]. Healthcare workers (HCWs) and people with underlying chronic conditions are at higher risk and should receive at least three vaccine doses and regular booster shots to protect against future COVID-19 variants [13]. A study conducted in Qatar and Israel showed that receiving a third booster dose significantly reduced illness by around 50% and lowered mortality and severe complications by more than 70% compared to those who received only two vaccine doses [14,15,16,17,18]. This strategy has been adopted as policy in many countries based on WHO recommendations, which include transitioning from a bivalent to a monovalent XBB-derived COVID-19 vaccine [11,12,19,20]. 

A meta-analysis revealed that the willingness rate of uptake of COVID-19 booster vaccines is high, reaching 81%. However, healthcare workers show a lower willingness rate at 66% compared to the general population, and willingness is notably lowest among Southeast Asians, with only 52%, a rate similar to the level of acceptance observed during the early COVID-19 vaccine rollout in Thailand [21,22]. Factors influencing acceptance of booster vaccines include increasing age, having chronic diseases such as cancer, trust in vaccine efficacy, history of receiving seasonal influenza vaccine, positive attitude towards vaccination, confidence in vaccine safety, recommendation by healthcare personnel, access to adequate vaccine information, belief that healthcare personnel are at high risk of COVID-19 infection, belief in the severity of COVID-19, being a healthcare professional, and not having contracted COVID-19 previously [23,24,25,26]. On the contrary, factors contributing to vaccine refusal include experiencing severe adverse reactions after prior vaccination and lack of belief in vaccine efficacy [23,24], especially among individuals who experienced breakthrough infections after vaccination, which doubled the propensity for vaccine refusal [21,27]. Vaccine hesitancy (VH) remains a significant challenge. About 44% of American healthcare workers who had not yet received a booster dose were concerned about potential side effects, citing the rapid development of the vaccines as a key reason for their hesitation (27%) and low perceived risk of infection (27%) [28]. While patient concerns were related to the necessity for a vaccine (64%), safety (60%) and vaccine effectiveness (54%) [28].

Currently, many countries have implemented policies for administering a booster dose now that COVID-19 has become an endemic disease, with most recommending the monovalent COVID-19 XBB-derived vaccine [29]. Information on vaccine willingness among healthcare professionals and vulnerable individuals remains essential due to the ongoing outbreaks of the Omicron variants and the potential emergence of new variants. This study aims to explore willingness, perceptions, and associated factors regarding COVID-19 booster doses among Thai healthcare workers and high-risk individuals during the post-pandemic era. Understanding the demand for booster doses and the factors influencing the willingness to accept a vaccine is crucial for policymakers to inform targeted strategies and health promotion activities aimed at high-risk groups. These efforts could improve vaccination uptake, enhance the protection of these key populations, and support broader public health goals.

## 2. Materials and Methods

### 2.1. Setting and Study Design

**Figure 1:**
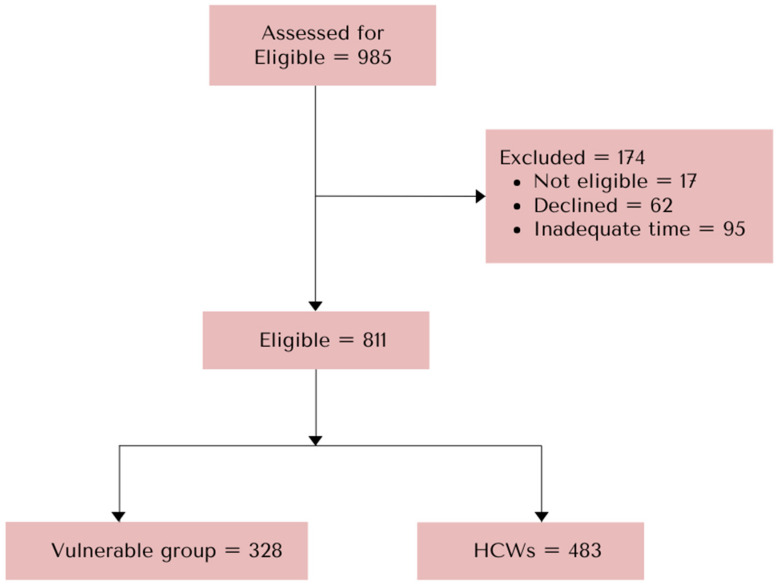
Flow diagram of participants’ enrollment.

This cross-sectional study took place in Chiang Mai, Northern Thailand, between October 2023 and May 2024. Participants included HCWs at Maharaj Nakorn Chiang Mai, which is a medical school and tertiary care facility, and patients seeking treatment for chronic medical conditions (Figure 1). In this study, the vulnerable population was defined as adults with medical conditions including obesity, diabetes, lipid profile, cardiovascular, chronic lung disease, asthma, chronic kidney disease, and immunocompromised status who are at a higher risk of severe illness from COVID-19. HCWs were recruited for the study when they visited the health promotion unit for vaccinations or health check-ups. Vulnerable populations were invited to participate during their outpatient visits to the Internal Medicine Department or during health check-ups at Maharaj Nakorn Chiang Mai Hospital. A trained research team assisted participants and asked for their consent to join the study and complete the questionnaire. The study included Thai adults aged 20 and above, encompassing HCWs, elderly individuals, and adults with chronic pulmonary diseases, cardiovascular diseases, chronic kidney disease, obesity, cancer, and diabetes mellitus. Potential participants who had received a monovalent XBB-derived booster vaccine were excluded. The study utilized a questionnaire divided into three sections: sociodemographic information, attitudes towards COVID-19 disease and COVID-19 booster vaccines, and factors influencing willingness to accept COVID-19 vaccine boosters. The questionnaire was developed based on prior research [22] and underwent face validity testing through a pilot study involving 50 individuals, which confirmed its reliability and validity as assessed by experts. Sample size calculations were based on an estimated willingness to vaccinate among those with chronic medical conditions (P_1_ = 0.66) and healthcare workers (P_2_ = 0.52) [21], with an alpha error of 0.05 and a beta error of 0.05. The calculated sample size was determined to be 775 individuals per group, and accounting for a 5% dropout rate, a total of 813 participants were needed for both groups.

### 2.2. Questionnaire

The research gathered demographic information from participants, encompassing details such as age, gender, occupation, education level, presence of chronic medical conditions, symptoms experienced during the most recent COVID-19 infection, categorized as mild (no medication needed), moderate (requiring medication), or severe (requiring hospitalization), history of COVID-19 among friends or family member, history of self-reported long COVID-19 (symptoms persisting for at least 3 months after SARS-CoV-2 infection), history of COVID-19 diagnosis, history of COVID-19 vaccination including the number of previous COVID-19 vaccinations, uptake of the bivalent COVID-19 vaccine, and history of flu vaccination during the last season. Additionally, participants’ perceptions towards COVID-19 were assessed, including perceived risk of future infection, perception of disease severity, the impact of COVID-19 on daily life and work, and willingness to receive a booster vaccine. This assessment also explored concerns related to vaccine side effects, safety, and efficacy. Perception was assessed using a five-level Likert scale, ranging from the lowest degree (1) to the highest degree (5). Responses of “Strongly agree” and “Agree” were considered as indicating a high perceived risk, while “Neither agree nor disagree”, “Disagree”, and “Strongly disagree” were categorized as reflecting a low perceived risk. The questionnaire was adapted from a previous study on vaccine acceptance and attitudes among the Thai population, with a validity score greater than 0.5 [22]. Of 811 overall participants, the attitude assessment demonstrated an excellent internal consistency in both Cronbach’s α (0.82; 95% CI 0.80–0.85). We also performed a subgroup analysis of vulnerable and healthcare worker groups to ensure the reliability of the assessment, which were 0.83 (95% CI 0.79–0.87) and 0.82 (95% CI 0.79–0.85), respectively.

### 2.3. Statistical Analysis

All statistical analyses were conducted using the STATA statistical software program (Stata Corp. 2023, Stata Statistical Software: Release 18, Stata Corp LLC, College Station, TX, USA). Descriptive statistics, including percentage, mean, and standard deviation (SD), were used to evaluate quantitative data from the questionnaire. Independent student T-tests and chi-square tests were used to analyze the differences in demographic data between the two groups. A multivariable logistic regression was analyzed to determine the independent determinants associated with COVID-19 vaccine willingness. Adjusted odds ratios (aOR) and 95% confidence intervals (95% CI) were presented as outcome parameters, which represented the magnitude of association between potential determinants with COVID-19 vaccine willingness. The statistical significance was set at a two-sided *p*-value below 0.05. The potential determinants, which were considered to explore in a multivariable logistic regression, were body mass index, participant groups (vulnerable and healthcare workers groups), chronic medical conditions, history of COVID-19 infection and vaccination, and attitude toward COVID-19 disease and COVID-19 vaccination. Other sociodemographic variables, including age, gender, education level, and marital status, were entered into the model as the potential confounders.

### 2.4. Ethical Considerations 

The informed consent, questionnaires, and study protocol received approval from the Research Ethics Committee of the Faculty of Medicine at Chiang Mai University (Ethical Approval Number: COM-2566-0404). Verbal informed consent was obtained from each participant before they joined the study, and participants were given the option to skip questions or withdraw their consent at any time.

## 3. Results

In this study, 811 participants were enrolled and divided into two groups: 328 in the vulnerable group and 483 HCWs (Table 1). Among the vulnerable group, the mean age was 52.7 years (SD = 16.5), with 61.3% (*n* = 201) being female, 15.2% (*n* = 50) had a body weight greater than 100 kg, 48.2% (*n* = 158) had a bachelor’s degree or higher education, and 57.9% (*n* = 190) were married. Regarding health conditions, 65.2% (*n* = 214) had cardiovascular diseases, 49.1% (n = 161) had hypertension, 41.8% (*n* = 137) had diabetes mellitus (DM), 35.7% (*n* = 117) had high cholesterol levels, 6.1% (*n* = 20) had chronic kidney disease, and 8.5% (*n* = 28) had cerebrovascular disease. With regard to COVID-19, 46.6% (n = 153) had a history of COVID-19 infection, with 10.7% (*n* = 35) experiencing mild symptoms and 32.9% (*n* = 108) having moderate symptoms that required medication; 14% (n = 46) reported long COVID-19 symptoms, and 37.8–50.3% (*n* = 124–165) had friends or family members who contracted COVID-19. Among the participants, 74.1% (n = 243) had their last COVID-19 infection more than one year after vaccination. In terms of vaccination, 91.1% (*n* = 297) had received COVID-19 vaccinations, with an average of three shots (IQR 2–4), with 10.4% (*n* = 34) receiving the bivalent COVID-19 vaccine. Side effects from the COVID-19 vaccine were reported by 26.2% (*n* = 86), with 25.3% (*n* = 83) experiencing mild symptoms such as pain at the injection site and low-grade fever. Additionally, 48% (*n* = 157) had received a flu shot within the past year, and only 17.7% (*n* = 58) had a history of vaccine refusal. Around 45% (*n* = 147) were willing to receive the monovalent XBB-derived vaccine if it became available.

Among the HCWs group, the mean age was 42.2 years (SD = 13.0), with 85.3% (*n* = 412) being female. 8.9% (*n* = 43) had a body weight greater than 100 kg, 72.9% (*n* = 352) had a bachelor’s degree or higher education, 53.8% (*n* = 260) were single, and 39.1% (*n* = 189) were married. 32.9% (*n* = 267) were nurses, followed by medical assistants (21.0%, *n* = 170) and doctors (4.8%, *n* = 39). 17.3% (*n* = 140) provided direct care for COVID-19 patients, and 40.9% (*n* = 332) were involved in caring for COVID-19 patients. Regarding health conditions, 12.2% (*n* = 59) had hypertension, 3.9% (*n* = 19) had DM, 8.5% (*n* = 41) had high cholesterol levels, 1.2% (*n* = 6) had chronic kidney disease, and 2.7% (*n* = 13) had cerebrovascular disease. With regard to COVID, 68.5% (*n* = 331) had a history of COVID-19 infection, with 20.9% (*n* = 101) experiencing mild symptoms and 46.6% (*n* = 225) having moderate symptoms that required medication; 24.2% (*n* = 117) reported long COVID-19 symptoms and 72.7–81.8% (*n* = 351–395) had friends or family members who contracted COVID-19. Among the participants, 70.2% (*n* = 339) had their last COVID-19 infection more than one year after vaccination. In terms of vaccination, 98.6% (*n* = 476) had received COVID-19 vaccinations, with an average of four shots (IQR 3–5); 32.1% (*n* = 155) had received the bivalent COVID-19 vaccine. Side effects from the COVID-19 vaccine were reported by 43.5% (*n* = 210), who experienced mild symptoms such as pain at the injection site and low-grade fever. Additionally, 75.4% (*n* = 364) had received a flu shot within the past year, and only 37.9% (*n* = 183) had a history of vaccine refusal. Around 43.3% (*n* = 209) were willing to receive the monovalent XBB-derived vaccine if it became available.

In Table 2, the attitudes toward COVID-19 and COVID-19 booster vaccine with monovalent XBB-derived are compared between the two groups. The results show that vulnerable individuals perceive a significantly lower risk of contracting COVID-19 in the future (83.5% vs. 59.6%, *p* < 0.001) but believe they have a significantly low risk of severe illness if infected (63.7% vs. 69.6%, *p* = 0.032). Both groups reported a similar low risk of COVID-19 impacting their daily lives (60.4.% vs. 62.9%, *p* = 0.380). When considering the impact of COVID-19 on work, vulnerable individuals felt more at a low-risk level compared to healthcare workers (59.8% vs. 51.6%, *p*-value = 0.030). Concerns about receiving a booster vaccine were similar between the groups (33.5% vs. 32.3%, *p* = 0.86), as were concerns about the side effects of booster vaccines (41.2% vs. 37.3%, *p* = 0.250). However, vulnerable individuals were significantly more likely to believe that booster vaccines are safe (29.3% vs. 20.7%, *p* = 0.009). Both groups had similar levels of belief that booster vaccines would prevent new strains of COVID-19 (23.8% vs. 18.6%, *p* = 0.095) and severe infection from new variants (32.9% vs. 29.8%, *p* = 0.300).

In Table 3, the factors influencing the willingness of vulnerable individuals and healthcare workers to accept a COVID-19 booster with the monovalent XBB-derived vaccine after the pandemic era are shown. Among the vulnerable group, 78.3% (*n* = 256) made the decision to acquire vaccination on their own, followed by 48.9% (*n* = 160) who were influenced by their friends and family members. In contrast, almost 90% (*n* = 431) of healthcare workers made the decision independently. Both groups had similar concerns about the monovalent XBB-derived booster vaccine (73.1% vs. 67.6%, *p* = 0.097), side effects (59.3% vs. 60.0%, *p* = 0.860), vaccine efficacy (36.4% vs. 39.6%, *p* = 0.350), and research on the vaccine (60.6% vs. 64.7%, *p* = 0.230). However, they differed significantly in terms of safety concerns (23.9% vs. 33.4%, *p* = 0.003). Regarding sources of information about the vaccine, the vulnerable group primarily relied on friends (53.5%, *n* = 175), the internet (38.8%, *n* = 127), and healthcare workers (33.9%, *n* = 111). 

Seven factors were associated with the willingness to receive the COVID-19 monovalent XBB-derived vaccine. Individuals with a body weight greater than 100 kg were 2.46 times more willing (95% CI: 1.41–4.31, *p* = 0.002). Each additional previous COVID-19 vaccination increased willingness by 1.15 times (95% CI: 1.03–1.29, *p* = 0.014). A history of receiving a flu vaccine in the past year increased willingness by 2.07 times (95% CI: 1.40–3.06, *p* < 0.001). Attitude towards COVID-19 impacting daily life increased willingness by 1.79 times (95% CI: 1.03–3.14, *p* = 0.040), and attitude towards COVID-19 impacting work life increased willingness by 1.74 times (95% CI: 1.02–2.95, *p* = 0.042). Belief in the safety of booster vaccines increased willingness by 1.85 times (95% CI: 1.11–3.07, *p* = 0.017), and belief that boosters will prevent severe infection from new variants increased willingness by 2.65 times (95% CI: 1.57–4.46, *p* < 0.001). Three factors were associated with hesitancy toward the COVID-19 monovalent XBB-derived vaccine. Healthcare workers were 42% less likely to be hesitant compared to the vulnerable group (aOR 0.58, 95% CI: 0.34–0.98, *p* = 0.040). Individuals with a history of vaccine refusal were 44% less likely to be willing compared to those without a history of refusal (aOR 0.56, 95% CI: 0.37–0.84, *p* = 0.005). Additionally, those concerned about receiving booster vaccines were 80% less likely to be willing compared to those who were not concerned (a OR 0.20, 95% CI: 0.11–0.36, *p* < 0.001). (Table 4).

## 4. Discussion

To the best of our knowledge, this was the first study conducted in Thailand to evaluate the willingness among healthcare workers and vulnerable patients post-pandemic to receive the monovalent XBB-derived COVID-19 booster vaccine. The willingness to receive the monovalent XBB-derived COVID-19 booster vaccine was 45.0% among vulnerable individuals and 43.3% among healthcare workers. Factors associated with a higher likelihood of getting the booster in the post-pandemic period included having a body weight over 100 kg, receiving a flu vaccine in the past year, perceiving that COVID-19 significantly affected daily life and work, believing in the safety of the booster, and trusting that the booster would prevent severe illness from new variants.

In our study, the willingness to receive a COVID-19 vaccine post-pandemic was around 44% among vulnerable groups and HCWs. This rate is notably lower than findings from a meta-analysis conducted during the pandemic up to June 2022, which reported an intention to receive a booster dose of 79% (95% CI 72–85%) among the general population and 66% (95% CI 58–74%) among HCWs [21]. Furthermore, our findings also show lower levels of willingness compared to studies conducted during the pandemic in 2021 among HCWs in Nepal, Vietnam, and Hong Kong, which reported willingness rates of 95.4%, 90.6%, and 54.4%, respectively [30]. In 2022, willingness to receive a booster dose among Chinese HCWs was reported at 76.4% [24]. Interestingly, our results are consistent with a study of the general population in Hong Kong during a period of low COVID-19 incidence (December 2021-January 2022), which found that only 42.2% intended to receive a booster dose [31]. This suggests a declining trend in the willingness to obtain a COVID-19 booster, and perhaps this study was conducted later or a longer time after the peak of the epidemic. Despite fluctuating willingness, actual uptake rates post-pandemic may be even lower. For example, a meta-analysis found that while the intention to receive a booster was around 79% among the general population, actual uptake was as low as 31% in some studies, particularly among those who expressed hesitancy or willingness [21,23,32]. These findings underscore the need for targeted efforts to emphasize the World Health Organization’s recommendation that high-priority groups—including adults with co-morbidities and healthcare workers in direct contact with patients—should receive booster doses to maintain protection [33].

In our study, 83.5% of the vulnerable population and 59.6% of HCWs believed they were at low risk of contracting COVID-19 in the future. Additionally, 63.7% to 69.6% thought the disease would have low severity, 60.4% to 62.9% expected it to have minimal impact on their daily lives, and 51.6% to 59.8% anticipated little impact on their work life post-pandemic. These results are quite different compared to previous studies, systematic reviews, and research conducted in Thailand during the pandemic, which showed high-risk awareness, fear, and anxiety in the general population. During that time, society strictly adhered to preventive measures and daily life was greatly affected [22,34]. However, HCWs, especially those on the front lines and in direct contact with patients, may have had a higher sense of risk compared to the general population, largely due to a lack of adequate infection control measures, including protective gear and supportive facilities [35]. Our findings are also in alignment with a study from the U.S., where people’s perception of risk decreased over time as they gained more experience regarding COVID-19 [36]. The slow rollout of vaccines, along with reduced media coverage and fewer public health messages, may also have contributed to this lower sense of risk [36,37]. Although people’s perception of risk can change over time, the risk of getting COVID-19 is never zero. It is important to continue reminding the public, especially vulnerable groups and HCWs, to take preventive actions to protect themselves from the disease.

In our study, 66.5–67.7% of participants expressed low levels of concern about receiving a booster vaccine post-pandemic, though 37.3–41.2% were highly concerned about potential side effects. Interestingly, 70.4–79.3% believed the booster vaccine was safe. However, almost 81.4% of HCWs and the vulnerable population doubted the booster’s effectiveness in preventing infection or severe illness from new COVID-19 variants. Social media and online platforms play a significant role in spreading both accurate information and misinformation. This is particularly true for anti-vaccine content, which often mimics the appearance of news or scientific reports, contributing to vaccine hesitancy. To effectively assess information, it is crucial to adopt various strategies, including fact-checking, evaluating the credibility of sources, and enhancing policies aimed at preventing the spread of misinformation [38,39]. This is similar to findings from a study among U.S. HCWs, where 63.6% were concerned about the vaccine being ineffective against new strains [40]. In a similar study of HCWs in Italy, 54.6% of those who planned not to acquire a booster believed it offered no protection against emerging variants, and 27% were concerned about side effects [25]. These attitudes could be related to breakthrough infections and waning immunity in fully vaccinated individuals [41]. The effectiveness of the XBB-derived vaccine against infection was 52.2% (95% CI: 44.6–58.7%) after 4 weeks, dropping to 20.4% (95% CI: 6.2–32.5%) after 20 weeks. Its effectiveness against hospitalization was 57.1% (95% CI: 40.4–69.2%) after 10 weeks for Omicron subvariants [42]. To successfully promote vaccination programs, enhancing perceptions of benefits alone may not be enough; the emphasis on vaccine safety, however, could be crucial in reducing vaccine hesitancy [43]. Some potential strategies include increasing health literacy campaigns to help vaccine recipients understand the importance of vaccination. Strengthening community engagement through social initiatives and improving healthcare workers’ communication and education skills are also key [44]. Additionally, the promotion of altruistic reasons and the highlighting of non-health benefits—like the ability to travel safely—could further encourage vaccine uptake [45,46]

Our study found that factors such as receiving additional COVID-19 vaccines (1.2 times more likely), having a flu shot in the past year (2.1 times more likely), concerns about the impact of COVID-19 on daily and work life (1.8 times more likely), and beliefs about the safety (1.8 times more likely) and effectiveness against severe disease (2.7 times more likely) significantly increased the willingness to obtain booster vaccination in the post-pandemic era. These findings are in alignment with previous research from both Asian and Western countries, as well as systematic reviews, which also highlight the connection between receiving the flu vaccine in the previous season, confidence in vaccine safety, and COVID-19 booster uptake [31,47,48,49]. This suggests that future annual COVID-19 vaccine booster campaigns targeting healthcare workers (HCWs) and vulnerable populations could benefit from emphasizing the safety and effectiveness of vaccines in preventing severe disease, as well as how COVID-19 can affect everyday life and work life. These campaigns could initially focus on those who have already received the flu vaccine or prior COVID-19 vaccinations, using them as trusted sources of information. Coupled with healthcare provider guidance, this approach could help increase vaccine uptake, combat misinformation, and reduce vaccine hesitancy in the post-pandemic era.

Numerous international studies have reported lower vaccine uptake during or shortly after the peak of the outbreaks. What sets this study apart is its focus on high-risk populations, particularly individuals with chronic medical conditions-a group that has been underrepresented in prior research. Additionally, this is the first study to gather data from various groups of healthcare workers and vulnerable individuals with underlying medical conditions in tertiary care settings. Uniquely, it was conducted after COVID-19 transitioned to an endemic phase and targeted the monovalent XBB-derived vaccine rather than other monovalent or bivalent COVID-19 vaccines. The findings could help inform practical strategies for the annual administration of COVID-19 vaccines, ensuring continued protection for high-risk populations in the post-pandemic era. This study has several limitations. The cross-sectional design does not permit the establishment of causal relationships between the identified determinants and the willingness to receive a COVID-19 booster in the post-pandemic era. Additionally, the study used convenience sampling at a single center, with most participants being doctors, nurses, and patients in tertiary care settings. This limits the generalizability of the findings to other populations or settings. The distribution of the questionnaire might also have attracted participants with pre-existing biases, either pro-vaccine or anti-vaccine, which could have influenced the results. Conducting the study during the endemic phase also limits its ability to compare vaccine uptake patterns across different phases of the pandemic. Future research should adopt a better sampling strategy and aim to include a more diverse sample, including other allied healthcare workers and individuals with co-morbidities. Expanding the study to multiple centers or conducting it at the national level would provide a more comprehensive understanding of vaccine willingness among Thai citizens. This could help in the design of more effective vaccine rollout campaigns for healthcare workers and vulnerable populations, ensuring better protection against COVID-19.

## 5. Conclusions

COVID-19 vaccine willingness is relatively low (44%) among HCWs and vulnerable populations in the post-pandemic era. This reduction likely stems from reduced perceived risk, disease severity, impacts on daily and work life, and confidence in the boosters’ effectiveness. Those who trust the boosters’ safety and effectiveness received a flu vaccine, never refused vaccination, and recognized that COVID-19’s impact may drive vaccine booster uptake. These individuals could serve as key advocates to encourage their peers, reduce vaccine hesitancy, and combat misinformation, helping to protect both themselves and their communities in the post-pandemic era. Policymakers could implement more effective strategies and health promotion activities targeted at high-risk groups.

## Figures and Tables

**Table 1:** Sociodemographic and COVID-19-related Information among Thai Healthcare Workers (HCWs) and the Vulnerable Population.

Sociodemographic	Total *n* (%)	Vulnerable Population *n* (%)	Health Care Worker *n* (%)	*p*-Value
Age in years, mean (SD)	46.5 (15.4)	52.7 (16.5)	42.2 (13.0)	<0.001 **
Female	613 (75.6%)	201 (61.3%)	412 (85.3)	<0.001 **
Body weight ≥ 100 kg	93 (11.5%)	50 (15.2%)	43 (8.9)	0.005 *
Education level				<0.001 **
Primary school	69 (8.5%)	65 (19.8%)	4 (0.8%)	
Secondary school	232 (28.6%)	105 (32.0%)	127 (26.3%)	
Bachelor’s degree	404 (49.8%)	117 (35.7%)	287 (59.4%)	
Master’s degree and above	106 (13.1%)	41 (12.5%)	65 (13.5%)	
Chronic medical condition				
Cardiovascular	308 (38.0%)	214 (65.2%)	94 (19.5%)	<0.001 **
Hypertension	220 (27.1%)	161 (49.1%)	59 (12.2%)	<0.001 **
Diabetes mellitus	156 (19.2%)	137 (41.8%)	19 (3.9%)	<0.001 **
Dyslipidemia	158 (19.5%)	117 (35.7%)	41 (8.5%)	<0.001 **
Chronic Obstructive Pulmonary Disease	60 (7.4%)	24 (7.3%)	36 (7.5%)	0.94
Asthma	20 (2.5%)	13 (4.0%)	7 (1.4%)	0.023 *
Chronic kidney disease	26 (3.2%)	20 (6.1%)	6 (1.2%)	<0.001 **
Cerebrovascular	41 (5.1%)	28 (8.5%)	13 (2.7%)	<0.001 **
Cancer	11 (1.4%)	8 (2.4%)	3 (0.6%)	0.028 *
History of COVID-19 infection				
Yourself	484 (59.7%)	153 (46.6%)	331 (68.5)	<0.001 **
Friends	516 (63.6%)	165 (50.3%)	351 (72.7%)	<0.001 **
Family members	519 (64.0%)	124 (37.8%)	395 (81.8%)	<0.001 **
Issues with long COVID-19	163 (20.1%)	46 (14.0%)	117 (24.2%)	<0.001 **
History of COVID-19 vaccine	774 (95.4%)	298 (90.9%)	476 (98.6%)	<0.001 **
Number of previous COVID-19 vaccination	4.0 (3.0–5.0)	3.0 (2.0–4.0)	4.0 (3.0–5.0)	<0.001 **
Received the Bivalent COVID-19 vaccine	189 (23.3%)	34 (10.4%)	155 (32.1%)	<0.001 **
Any side effect from COVID-19 vaccine	300 (37.0%)	86 (26.2%)	214 (44.3%)	<0.001 **
History of flu vaccine last year	521 (64.2%)	157 (47.9%)	364 (75.4%)	<0.001 **
History of any vaccine refusal	241 (29.7%)	58 (17.7%)	183 (37.9%)	<0.001 **
Will receive the latest COVID-19 vaccine	356 (44.0%)	147 (44.8%)	209 (43.3%)	0.640

* *p*-value < 0.05, ** *p*-value < 0.001.

**Table 2:** Attitudes toward COVID-19 Disease and COVID-19 Vaccination Among Thai Healthcare Workers (HCWs) and the Vulnerable Group.

Parameter	Perceived Risk	Vulnerable Population *n* (%)	Healthcare Workers *n* (%)	*p*-Value
The risk level for contracting COVID-19 in the future	Low	274 (83.5%)	288 (59.6%)	<0.001 **
	High	51 (15.5%)	195 (40.4%)	
Severity level of COVID-19	Low severity	209 (63.7%)	336 (69.6%)	0.032 *
	High severity	116 (35.4%)	147 (30.4%)	
COVID-19 impacts your daily life	Low impact	198 (60.4%)	304 (62.9%)	0.380
	High impact	129 (39.3%)	179 (37.1%)	
COVID-19 impact your work life	Low impact	196 (59.8%)	249 (51.6%)	0.030
	High impact	131 (39.9%)	234 (48.4%)	
Concerns about receiving a booster vaccine	Low risk	217 (66.2%)	327 (67.7%)	0.86
	High risk	110 (33.5%)	156 (32.3%)	
Concerned about the side effects of booster vaccines	Low concern	192 (58.5%)	303 (62.7%)	0.250
	High concern	135 (41.2%)	180 (37.3%)	
Believe booster vaccines are safe	Low beliefs	231 (70.4%)	383 (79.3%)	0.009 *
	High beliefs	96 (29.3%)	100 (20.7%)	
Believe booster vaccines will prevent infection with new strains of COVID-19	Low beliefs	249 (75.9%)	393 (81.4%)	0.095
	High beliefs	78 (23.8%)	90 (18.6%)	
believe booster vaccines will prevent severe infection from new variants of COVID-19	Low beliefs	219 (66.8%)	339 (70.2%)	0.300
	High beliefs	108 (32.9%)	144 (9.8%)	

* *p*-value < 0.05, ** *p*-value < 0.001.

**Table 3:** Factors influencing COVID-19 Booster willingness among Thai Healthcare Workers (HCWs) and the Vulnerable Population.

Parameter	Total *n* (%)	Vulnerable Population *n* (%)	Healthcare Workers *n* (%)	*p*-Value
The biggest influence of getting the vaccine				
Yourself	687 (84.8%)	256 (78.3%)	431 (89.2%)	<0.001 **
Friend and Family member	283 (34.9%)	160 (48.9%)	123 (25.5%)	<0.001 **
Vaccine expert	168 (20.7%)	50 (15.3%)	118 (24.4%)	0.002 *
Government	95 (11.7%)	40 (12.2%)	55 (11.4%)	0.710
Community	37 (4.6%)	28 (8.6%)	9 (1.9%)	<0.001 **
Any concerns	565 (69.8%)	239 (73.1%)	326 (67.6%)	0.097
Side effect	483 (59.7%)	194 (59.3%)	289 (60.0%)	0.860
Efficacy	310 (38.3%)	119 (36.4%)	191 (39.6%)	0.350
Immunity	241 (29.8%)	87 (26.6%)	154 (32.0%)	0.100
Research on vaccine	510 (63.0%)	198 (60.6%)	312 (64.7%)	0.230
Safety	239 (29.5%)	78 (23.9%)	161 (33.4%)	0.003 *
Source of information				
Friend	444 (54.9%)	175 (53.5%)	269 (55.8%)	0.520
Internet	237 (29.3%)	127 (38.8%)	110 (22.8%)	<0.001 **
Healthcare Workers	197 (24.4%)	111 (33.9%)	86 (17.8%)	<0.001 **
Offline	160 (19.8%)	68 (20.8%)	92 (19.1%)	0.550

* *p*-value < 0.05, ** *p*-value < 0.001.

**Table 4:** Factors associated with COVID-19 vaccine willingness in the post-pandemic era among HCWs and this vulnerable population.

COVID-19 Vaccine Willingness	aORs	95% CI		*p*-Value
		UCI	LCI	
Sociodemographic				
Healthcare workers (reference: high-risk group)	0.58	0.34	0.98	0.040 *
Body weight > 100 kg.	2.46	1.41	4.31	0.002 *
Chronic medical conditions				
Respiratory diseases (reference: no)	1.63	0.82	3.21	0.161
Cardiovascular diseases (reference: no)	0.85	0.55	1.32	0.465
Chronic kidney disease (reference: no)	1.23	0.45	3.35	0.680
Cerebrovascular (reference: no)	0.71	0.29	1.69	0.434
Cancer (reference: no)	0.92	0.16	5.35	0.925
Diabetes mellitus (reference: no)	0.88	0.54	1.45	0.622
History of COVID-19 infection				
Yourself (reference: no)	1.29	0.74	2.22	0.368
Friends (reference: no)	1.16	0.71	1.90	0.548
Family member (reference: no)	1.28	0.80	2.04	0.309
Issues with Long COVID-19 (reference: no)	0.62	0.38	1.01	0.056
Number of COVID-19 vaccination	1.15	1.03	1.29	0.014 *
Have you received the (Bivalent) vaccine (reference: no)	1.07	0.69	1.66	0.750
Side effects from COVID-19 vaccine (reference: no)	0.72	0.49	1.05	0.088
History of flu vaccine last year (reference: no)	2.07	1.40	3.06	<0.001 *
History of any vaccine refusal (reference: no)	0.56	0.37	0.84	0.005 *
Attitude toward COVID-19 disease and COVID-19 vaccination (reference: low perceived)				
Risk levels for contracting COVID-19 in the future	1.43	0.94	2.18	0.092
Severity levels of COVID-19?	1.18	0.76	1.82	0.464
COVID-19 impacting your daily life	1.79	1.03	3.14	0.040 *
COVID-19 impacting your work life	1.74	1.02	2.95	0.042 *
Concerns about receiving a booster vaccine	0.20	0.11	0.36	<0.001 **
Concerned about the side effects of booster vaccines	0.71	0.42	1.20	0.201
Believe booster vaccines are safe	1.85	1.11	3.07	0.017 *
Believe booster vaccines will prevent infection with new strains of COVID-19	1.12	0.61	2.06	0.715
Believe booster vaccines will prevent severe infection from new variants of COVID-19	2.65	1.57	4.46	<0.001 **

Abbreviation: aOR, adjusted Odds Ratio; 95% CI, 95% confidence interval. aOR was obtained by a multivariable logistic regression model adjusted for age, gender, education levels, and marital status. * *p*-value < 0.05, ** *p*-value < 0.001.

## Data Availability

The original contributions presented in the study are included in the article. Further inquiries can be directed at the corresponding author.
